# (6,6′-Dimeth­oxy­biphenyl-2,2′-di­yl)bis(diphenyl­phosphane) *P*,*P*′-dioxide dihydrate

**DOI:** 10.1107/S1600536812005314

**Published:** 2012-02-24

**Authors:** Dongmei Dai, Lin Tang, Yanqing Gong

**Affiliations:** a Key Laboratory for Molecular Design and Nutrition Engineering, Ningbo Institute of Technology, Zhejiang University, Ningbo 315104, People’s Republic of China; b Zhejiang Pharmaceutical College, Ningbo 315100, People’s Republic of China; c State Key Laboratory of Bio-Organic and Natural Products Chemistry, Shanghai Institute of Organic Chemistry, CAS, 345 Lingling Road, Shanghai 200032, People’s Republic of People’s Republic of China

## Abstract

In the title compound, C_38_H_32_O_4_P_2_·2H_2_O, the dihedral angle between the meth­oxy­phenol rings is 84.11 (7)°. O—H⋯O hydrogen bonds connect the water mol­ecules of crystallization with the main mol­ecule.

## Related literature

For the synthesis of the title compound and its unsolvated crystal structure, see: Doherty *et al.* (2009 ▶). For similar structures, see: Meijboom (2011 ▶); Wang *et al.* (2011 ▶); Warsink *et al.* (2011 ▶).
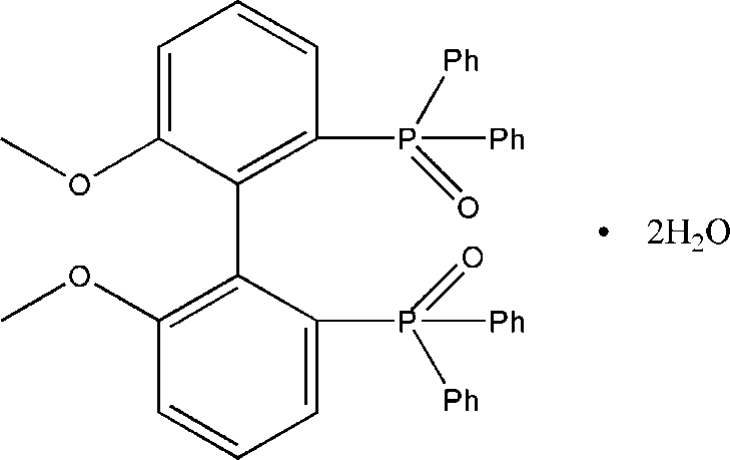


## Experimental

### 

#### Crystal data

C_38_H_32_O_4_P_2_·2H_2_O
*M* *_r_* = 650.61
Orthorhombic, 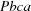

*a* = 13.108 (3) Å
*b* = 15.650 (3) Å
*c* = 33.967 (7) Å
*V* = 6968 (3) Å^3^
*Z* = 8
Cu *K*α radiation
μ = 1.50 mm^−1^
*T* = 296 K
0.23 × 0.10 × 0.10 mm

#### Data collection

Bruker APEXII CCD diffractometer
Absorption correction: multi-scan (*SADABS*; Bruker, 2009 ▶) *T*_min_ = 0.725, *T*_max_ = 0.865
31453 measured reflections
6197 independent reflections
4670 reflections with *I* > 2σ(*I*)
*R*_int_ = 0.042

#### Refinement

*R*[*F* ^2^ > 2σ(*F* ^2^)] = 0.044
*wR*(*F* ^2^) = 0.130
*S* = 1.03
6197 reflections
415 parameters
3 restraints
H-atom parameters constrained
Δρ_max_ = 0.20 e Å^−3^
Δρ_min_ = −0.29 e Å^−3^

### 

Data collection: *APEX2* (Bruker, 2009 ▶); cell refinement: *SAINT* (Bruker, 2009 ▶); data reduction: *SAINT*; program(s) used to solve structure: *SHELXS97* (Sheldrick, 2008 ▶); program(s) used to refine structure: *SHELXL97* (Sheldrick, 2008 ▶); molecular graphics: *SHELXTL* (Sheldrick, 2008 ▶); software used to prepare material for publication: *SHELXTL*.

## Supplementary Material

Crystal structure: contains datablock(s) I, global. DOI: 10.1107/S1600536812005314/hb6568sup1.cif

Structure factors: contains datablock(s) I. DOI: 10.1107/S1600536812005314/hb6568Isup2.hkl

Supplementary material file. DOI: 10.1107/S1600536812005314/hb6568Isup3.cml

Additional supplementary materials:  crystallographic information; 3D view; checkCIF report

## Figures and Tables

**Table 1:** Hydrogen-bond geometry (Å, °)

*D*—H⋯*A*	*D*—H	H⋯*A*	*D*⋯*A*	*D*—H⋯*A*
O1′—H1′*A*⋯O4	0.85	2.31	2.854 (4)	123
O2′—H2′*A*⋯O3	0.85	1.96	2.799 (3)	169

**Fig. 1.**
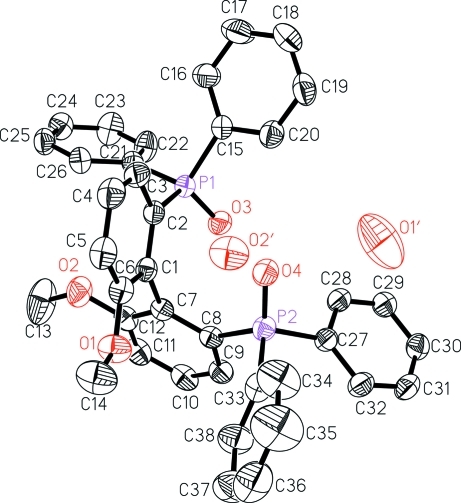
View of the molecule of (I) showing displacement ellipsoids drawn at the 30% probability level. Hydrogen atoms were omitted for clarity.

**Fig. 2.**
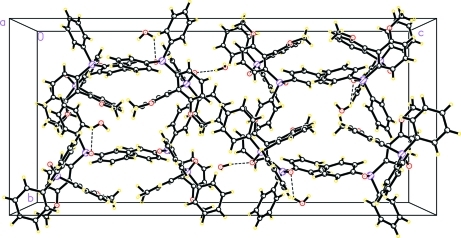
The packing of the title compound, viewed down the a axis. Dashed lines indicate the O—H···O hydrogen bonds.

## supplementary crystallographic information

### Comment

The title compound, (I) (Fig. 1), (6,6'-Dimethoxybiphenyl-2,2'-diyl)- bis(diphenylphosphine)dioxide dihydrate can be synthesized according to the procedure of Doherty *et al.*, (2009).

Compared with the structure of C_38_H_32_O_4_P_2_ (CCDC 756817), Doherty *et al.*, (2009), in which weak intermolecular C—H···O hydrogen bonds pull adjacent molecules closer, the interesting difference is the two solvent waters in the asymmetric unit, which form strong O—H···O hydrogen bonds with O atom of P═O,(Fig. 2). The waters take place of the adjacent bulky molecules to stabilize the crystal packing. As a result, the molecules pack in a different, much looser form in the crystal. And the calculated density of the crystal also confirms this point, 1.240 and 1.419 g cm^-3^ for the title compoud and the previous structure, respectively.

The whole structure exhibits as a dimer of triarylphosphorus oxide through C1—C7 covalent bond. The bond lengths [1.803 (2)–1.812 (2) Å] and angles[104.05 (10)–107.27 (11)°] of C_aryl_—P do not show large deviations from those observed in related structures (Meijboom, 2011; Wang, *et al.*, 2011; Warsink, *et al.*, 2011). Two methoxyphenyl rings locate almost perpendicular to each other, with a dihedral angle of 84.11(0.07)°.

### Experimental

The title compound was prepared according to the procedure of Doherty *et al.*, (2009) through double cycloaddition-elimination by using 1,4-bis-(diphenylphosphinoyl)buta-1,3-diyne and 1-methoxy-1,3-cycolhexadiene, heated with microwave in toluene solution. Colourless blocks were obtained from acetone/water(1:1) solution after about a week at room temperature.

### Refinement

The water H atoms were located in difference Fourier map and were then subsequently treated as riding atoms with O—H distances of 0.85 Å and *U*_iso_(H) = 1.5*U*_eq_(O). The locations of the water H atoms should be regarded as less certain than those of the other atoms. All non-solvent H atoms were placed in geometically idealized positions and constrained to ride on their parent atoms with C—H distances of 0.93 Å(0.96 for methyl group) and *U*_iso_(H) = 1.2(1.5 for CH3)*U*_eq_(C) for CH.

### Crystal data

**Table:**

C_38_H_32_O_4_P_2_·2H_2_O	*F*(000) = 2736
*M**_r_* = 650.61	*D*_x_ = 1.240 Mg m^−^^3^
Orthorhombic, *P**b**c**a*	Cu *K*α radiation, λ = 1.54178 Å
Hall symbol: -P 2ac 2ab	Cell parameters from 4670 reflections
*a* = 13.108 (3) Å	θ = 2.6–67.7°
*b* = 15.650 (3) Å	µ = 1.50 mm^−^^1^
*c* = 33.967 (7) Å	*T* = 296 K
*V* = 6968 (3) Å^3^	Block, colourless
*Z* = 8	0.23 × 0.10 × 0.10 mm

### Data collection

**Table:**

Bruker APEXII CCD diffractometer	6197 independent reflections
Radiation source: fine-focus sealed tube	4670 reflections with *I* > 2σ(*I*)
Graphite monochromator	*R*_int_ = 0.042
φ and ω scans	θ_max_ = 67.7°, θ_min_ = 2.6°
Absorption correction: multi-scan (*SADABS*; Bruker, 2009)	*h* = −15→12
*T*_min_ = 0.725, *T*_max_ = 0.865	*k* = −18→18
31453 measured reflections	*l* = −35→40

### Refinement

**Table:**

Refinement on *F*^2^	Primary atom site location: structure-invariant direct methods
Least-squares matrix: full	Secondary atom site location: difference Fourier map
*R*[*F*^2^ > 2σ(*F*^2^)] = 0.044	Hydrogen site location: inferred from neighbouring sites
*wR*(*F*^2^) = 0.130	H-atom parameters constrained
*S* = 1.03	*w* = 1/[σ^2^(*F*_o_^2^) + (0.066*P*)^2^ + 1.3879*P*] where *P* = (*F*_o_^2^ + 2*F*_c_^2^)/3
6197 reflections	(Δ/σ)_max_ = 0.001
415 parameters	Δρ_max_ = 0.20 e Å^−^^3^
3 restraints	Δρ_min_ = −0.29 e Å^−^^3^

### Special details

**Table:**

Geometry. All e.s.d.'s (except the e.s.d. in the dihedral angle between two l.s. planes) are estimated using the full covariance matrix. The cell e.s.d.'s are taken into account individually in the estimation of e.s.d.'s in distances, angles and torsion angles; correlations between e.s.d.'s in cell parameters are only used when they are defined by crystal symmetry. An approximate (isotropic) treatment of cell e.s.d.'s is used for estimating e.s.d.'s involving l.s. planes.
Refinement. Refinement of *F*^2^ against ALL reflections. The weighted *R*-factor *wR* and goodness of fit *S* are based on *F*^2^, conventional *R*-factors *R* are based on *F*, with *F* set to zero for negative *F*^2^. The threshold expression of *F*^2^ > σ(*F*^2^) is used only for calculating *R*-factors(gt) *etc*. and is not relevant to the choice of reflections for refinement. *R*-factors based on *F*^2^ are statistically about twice as large as those based on *F*, and *R*- factors based on ALL data will be even larger.

### Fractional atomic coordinates and isotropic or equivalent isotropic displacement parameters (Å^2^)

**Table:**

	*x*	*y*	*z*	*U*_iso_*/*U*_eq_	
P1	0.80685 (4)	0.18583 (3)	0.593688 (18)	0.05612 (16)	
P2	1.07260 (4)	0.30232 (4)	0.651596 (17)	0.05821 (17)	
O1	1.10418 (14)	0.07362 (13)	0.67804 (5)	0.0805 (5)	
O2	1.00948 (16)	0.01615 (12)	0.58155 (6)	0.0875 (6)	
O3	0.87621 (12)	0.24506 (10)	0.57242 (5)	0.0680 (4)	
O4	0.96975 (12)	0.30072 (10)	0.66970 (5)	0.0710 (4)	
C1	0.96504 (16)	0.12224 (12)	0.64144 (6)	0.0516 (5)	
C2	0.86030 (16)	0.13410 (12)	0.63672 (6)	0.0539 (5)	
C3	0.79402 (19)	0.10720 (15)	0.66641 (7)	0.0682 (6)	
H3A	0.7241	0.1153	0.6635	0.082*	
C4	0.8310 (2)	0.06900 (16)	0.69975 (8)	0.0751 (7)	
H4A	0.7858	0.0507	0.7191	0.090*	
C5	0.9343 (2)	0.05729 (16)	0.70501 (7)	0.0699 (6)	
H5A	0.9589	0.0320	0.7279	0.084*	
C6	1.00078 (18)	0.08347 (14)	0.67601 (6)	0.0597 (5)	
C7	1.04118 (15)	0.14815 (13)	0.61094 (6)	0.0515 (5)	
C8	1.09387 (15)	0.22572 (14)	0.61251 (6)	0.0532 (5)	
C9	1.16457 (18)	0.24536 (15)	0.58293 (7)	0.0641 (6)	
H9A	1.2000	0.2968	0.5838	0.077*	
C10	1.1816 (2)	0.18884 (17)	0.55269 (7)	0.0727 (7)	
H10A	1.2285	0.2025	0.5331	0.087*	
C11	1.1304 (2)	0.11233 (18)	0.55099 (7)	0.0728 (7)	
H11A	1.1422	0.0747	0.5303	0.087*	
C12	1.06147 (18)	0.09148 (15)	0.58007 (7)	0.0622 (6)	
C13	1.0309 (4)	−0.0473 (2)	0.55322 (12)	0.1502 (19)	
H13A	0.9883	−0.0962	0.5579	0.225*	
H13B	1.1013	−0.0637	0.5552	0.225*	
H13C	1.0176	−0.0251	0.5274	0.225*	
C14	1.1479 (3)	0.0481 (2)	0.71429 (9)	0.1078 (11)	
H14A	1.2206	0.0438	0.7114	0.162*	
H14B	1.1207	−0.0065	0.7219	0.162*	
H14C	1.1321	0.0896	0.7342	0.162*	
C15	0.69724 (17)	0.24298 (13)	0.61195 (7)	0.0627 (6)	
C16	0.5982 (2)	0.2192 (2)	0.60574 (14)	0.1231 (14)	
H16A	0.5844	0.1698	0.5915	0.148*	
C17	0.5185 (3)	0.2673 (3)	0.62022 (16)	0.152 (2)	
H17A	0.4517	0.2492	0.6163	0.183*	
C18	0.5367 (3)	0.3408 (2)	0.64014 (11)	0.1039 (10)	
H18A	0.4828	0.3735	0.6497	0.125*	
C19	0.6331 (3)	0.36566 (17)	0.64588 (9)	0.0855 (8)	
H19A	0.6459	0.4163	0.6593	0.103*	
C20	0.7139 (2)	0.31772 (15)	0.63230 (8)	0.0727 (6)	
H20A	0.7804	0.3360	0.6369	0.087*	
C21	0.75769 (17)	0.10246 (13)	0.56231 (6)	0.0573 (5)	
C22	0.7201 (2)	0.12554 (17)	0.52578 (8)	0.0844 (8)	
H22A	0.7222	0.1825	0.5181	0.101*	
C23	0.6794 (3)	0.0651 (2)	0.50058 (8)	0.0949 (9)	
H23A	0.6537	0.0815	0.4762	0.114*	
C24	0.6771 (2)	−0.01876 (18)	0.51153 (8)	0.0778 (7)	
H24A	0.6497	−0.0594	0.4946	0.093*	
C25	0.7145 (2)	−0.04304 (15)	0.54702 (8)	0.0721 (7)	
H25A	0.7129	−0.1003	0.5543	0.087*	
C26	0.7550 (2)	0.01716 (14)	0.57237 (7)	0.0670 (6)	
H26A	0.7809	−0.0002	0.5966	0.080*	
C27	1.09683 (17)	0.40435 (14)	0.62868 (7)	0.0587 (5)	
C28	1.02525 (18)	0.43283 (15)	0.60170 (7)	0.0668 (6)	
H28A	0.9701	0.3982	0.5951	0.080*	
C29	1.0353 (2)	0.51243 (16)	0.58446 (8)	0.0740 (7)	
H29A	0.9868	0.5313	0.5664	0.089*	
C30	1.1163 (2)	0.56346 (16)	0.59384 (8)	0.0738 (7)	
H30A	1.1224	0.6172	0.5824	0.089*	
C31	1.1881 (2)	0.53592 (16)	0.61996 (9)	0.0782 (7)	
H31A	1.2435	0.5707	0.6259	0.094*	
C32	1.17926 (19)	0.45643 (16)	0.63770 (8)	0.0726 (7)	
H32A	1.2284	0.4381	0.6556	0.087*	
C33	1.1719 (2)	0.28665 (16)	0.68767 (7)	0.0703 (6)	
C34	1.1509 (3)	0.3085 (3)	0.72569 (10)	0.1249 (13)	
H34A	1.0867	0.3293	0.7323	0.150*	
C35	1.2251 (5)	0.2998 (4)	0.75440 (12)	0.168 (2)	
H35A	1.2104	0.3166	0.7800	0.202*	
C36	1.3176 (4)	0.2678 (3)	0.74624 (13)	0.1293 (16)	
H36A	1.3661	0.2615	0.7660	0.155*	
C37	1.3394 (3)	0.2448 (3)	0.70872 (12)	0.1192 (13)	
H37A	1.4035	0.2230	0.7026	0.143*	
C38	1.2661 (3)	0.2537 (2)	0.67941 (9)	0.0958 (9)	
H38A	1.2814	0.2370	0.6538	0.115*	
O2'	0.9356 (2)	0.2718 (2)	0.49425 (8)	0.1491 (11)	
H2'A	0.9192	0.2565	0.5174	0.224*	
H2'B	0.9978	0.2869	0.4960	0.224*	
O1'	0.9143 (4)	0.4666 (3)	0.69732 (12)	0.228 (2)	
H1'A	0.9114	0.4391	0.6758	0.342*	
H1'B	0.9306	0.4285	0.7140	0.342*	

### Atomic displacement parameters (Å^2^)

**Table:**

	*U*^11^	*U*^22^	*U*^33^	*U*^12^	*U*^13^	*U*^23^
P1	0.0547 (3)	0.0467 (3)	0.0669 (4)	−0.0015 (2)	0.0027 (2)	0.0036 (2)
P2	0.0619 (3)	0.0572 (3)	0.0556 (3)	−0.0052 (2)	0.0050 (2)	−0.0018 (3)
O1	0.0752 (11)	0.1074 (14)	0.0588 (10)	0.0052 (10)	−0.0087 (8)	0.0174 (9)
O2	0.1042 (15)	0.0723 (11)	0.0862 (13)	−0.0095 (10)	0.0138 (10)	−0.0268 (10)
O3	0.0634 (9)	0.0622 (9)	0.0785 (11)	−0.0065 (7)	0.0018 (8)	0.0160 (8)
O4	0.0722 (10)	0.0677 (10)	0.0731 (10)	−0.0086 (8)	0.0224 (8)	−0.0119 (8)
C1	0.0609 (12)	0.0465 (10)	0.0474 (11)	−0.0029 (9)	0.0048 (9)	−0.0025 (9)
C2	0.0591 (12)	0.0454 (10)	0.0571 (12)	−0.0022 (9)	0.0062 (9)	−0.0018 (9)
C3	0.0646 (14)	0.0648 (14)	0.0753 (16)	−0.0039 (11)	0.0159 (11)	0.0025 (12)
C4	0.0916 (19)	0.0719 (15)	0.0618 (15)	−0.0090 (13)	0.0237 (13)	0.0038 (12)
C5	0.0956 (19)	0.0663 (14)	0.0479 (13)	−0.0063 (13)	0.0060 (12)	0.0021 (11)
C6	0.0712 (14)	0.0584 (13)	0.0497 (12)	−0.0036 (10)	0.0000 (10)	−0.0021 (10)
C7	0.0524 (11)	0.0572 (11)	0.0448 (11)	0.0051 (9)	−0.0008 (8)	0.0027 (9)
C8	0.0519 (12)	0.0597 (12)	0.0482 (11)	0.0046 (9)	0.0035 (8)	0.0033 (9)
C9	0.0623 (13)	0.0659 (13)	0.0642 (14)	0.0042 (11)	0.0123 (10)	0.0116 (11)
C10	0.0767 (16)	0.0872 (18)	0.0543 (14)	0.0201 (14)	0.0200 (11)	0.0167 (12)
C11	0.0863 (18)	0.0801 (17)	0.0522 (13)	0.0184 (14)	0.0109 (11)	−0.0026 (12)
C12	0.0686 (14)	0.0652 (13)	0.0528 (12)	0.0090 (11)	0.0026 (10)	−0.0033 (10)
C13	0.245 (6)	0.096 (3)	0.110 (3)	−0.033 (3)	0.049 (3)	−0.046 (2)
C14	0.105 (2)	0.150 (3)	0.0676 (18)	0.018 (2)	−0.0213 (16)	0.0263 (19)
C15	0.0613 (13)	0.0479 (11)	0.0788 (15)	−0.0004 (10)	0.0012 (11)	−0.0032 (11)
C16	0.0627 (18)	0.093 (2)	0.213 (4)	−0.0049 (15)	0.009 (2)	−0.073 (3)
C17	0.065 (2)	0.135 (3)	0.257 (6)	0.007 (2)	0.014 (3)	−0.096 (4)
C18	0.089 (2)	0.088 (2)	0.134 (3)	0.0257 (18)	0.0201 (19)	−0.020 (2)
C19	0.112 (2)	0.0569 (14)	0.088 (2)	0.0085 (15)	0.0185 (16)	−0.0100 (13)
C20	0.0800 (17)	0.0644 (14)	0.0738 (16)	−0.0074 (12)	0.0047 (12)	−0.0072 (12)
C21	0.0608 (13)	0.0540 (12)	0.0570 (12)	0.0011 (9)	0.0060 (10)	0.0006 (10)
C22	0.120 (2)	0.0645 (15)	0.0687 (16)	−0.0097 (15)	−0.0074 (15)	0.0094 (13)
C23	0.137 (3)	0.091 (2)	0.0572 (15)	−0.0121 (19)	−0.0136 (16)	0.0031 (15)
C24	0.0936 (19)	0.0745 (17)	0.0652 (16)	−0.0039 (14)	0.0093 (13)	−0.0166 (13)
C25	0.0838 (17)	0.0520 (13)	0.0804 (17)	0.0009 (11)	0.0039 (13)	−0.0065 (12)
C26	0.0778 (16)	0.0547 (12)	0.0686 (15)	0.0027 (11)	−0.0018 (12)	0.0009 (11)
C27	0.0562 (12)	0.0574 (12)	0.0626 (13)	−0.0039 (9)	0.0022 (10)	−0.0029 (10)
C28	0.0606 (14)	0.0619 (13)	0.0779 (16)	−0.0073 (11)	−0.0072 (11)	0.0004 (12)
C29	0.0722 (16)	0.0658 (15)	0.0841 (18)	0.0024 (12)	−0.0062 (12)	0.0043 (13)
C30	0.0782 (17)	0.0580 (13)	0.0851 (18)	−0.0042 (12)	0.0047 (13)	0.0057 (13)
C31	0.0718 (16)	0.0683 (15)	0.094 (2)	−0.0198 (12)	−0.0023 (14)	0.0010 (14)
C32	0.0649 (15)	0.0703 (15)	0.0827 (17)	−0.0114 (11)	−0.0117 (12)	0.0052 (13)
C33	0.0855 (18)	0.0676 (14)	0.0579 (14)	−0.0066 (12)	−0.0076 (12)	0.0045 (11)
C34	0.139 (3)	0.172 (4)	0.0640 (19)	0.026 (3)	−0.0141 (19)	−0.015 (2)
C35	0.192 (5)	0.242 (6)	0.071 (2)	0.030 (5)	−0.041 (3)	−0.024 (3)
C36	0.163 (4)	0.127 (3)	0.098 (3)	−0.025 (3)	−0.065 (3)	0.027 (2)
C37	0.103 (3)	0.139 (3)	0.116 (3)	0.000 (2)	−0.041 (2)	0.022 (3)
C38	0.091 (2)	0.120 (2)	0.0771 (18)	0.0091 (18)	−0.0196 (15)	0.0020 (17)
O2'	0.147 (2)	0.198 (3)	0.1023 (18)	0.006 (2)	0.0169 (16)	0.027 (2)
O1'	0.287 (5)	0.217 (4)	0.179 (3)	0.099 (4)	0.046 (3)	−0.055 (3)

### Geometric parameters (Å, º)

**Table:**

P1—O3	1.4859 (16)	C18—C19	1.337 (4)
P1—C15	1.802 (2)	C18—H18A	0.9300
P1—C21	1.804 (2)	C19—C20	1.377 (4)
P1—C2	1.812 (2)	C19—H19A	0.9300
P2—O4	1.4819 (16)	C20—H20A	0.9300
P2—C33	1.804 (3)	C21—C26	1.378 (3)
P2—C27	1.804 (2)	C21—C22	1.383 (3)
P2—C8	1.810 (2)	C22—C23	1.383 (4)
O1—C6	1.366 (3)	C22—H22A	0.9300
O1—C14	1.416 (3)	C23—C24	1.364 (4)
O2—C12	1.363 (3)	C23—H23A	0.9300
O2—C13	1.411 (4)	C24—C25	1.356 (4)
C1—C2	1.395 (3)	C24—H24A	0.9300
C1—C6	1.402 (3)	C25—C26	1.383 (3)
C1—C7	1.495 (3)	C25—H25A	0.9300
C2—C3	1.396 (3)	C26—H26A	0.9300
C3—C4	1.369 (4)	C27—C28	1.385 (3)
C3—H3A	0.9300	C27—C32	1.388 (3)
C4—C5	1.379 (4)	C28—C29	1.383 (3)
C4—H4A	0.9300	C28—H28A	0.9300
C5—C6	1.377 (3)	C29—C30	1.366 (4)
C5—H5A	0.9300	C29—H29A	0.9300
C7—C8	1.398 (3)	C30—C31	1.364 (4)
C7—C12	1.399 (3)	C30—H30A	0.9300
C8—C9	1.401 (3)	C31—C32	1.387 (3)
C9—C10	1.374 (3)	C31—H31A	0.9300
C9—H9A	0.9300	C32—H32A	0.9300
C10—C11	1.374 (4)	C33—C34	1.364 (4)
C10—H10A	0.9300	C33—C38	1.368 (4)
C11—C12	1.378 (3)	C34—C35	1.384 (6)
C11—H11A	0.9300	C34—H34A	0.9300
C13—H13A	0.9600	C35—C36	1.341 (6)
C13—H13B	0.9600	C35—H35A	0.9300
C13—H13C	0.9600	C36—C37	1.355 (6)
C14—H14A	0.9600	C36—H36A	0.9300
C14—H14B	0.9600	C37—C38	1.391 (4)
C14—H14C	0.9600	C37—H37A	0.9300
C15—C16	1.367 (4)	C38—H38A	0.9300
C15—C20	1.376 (3)	O2'—H2'A	0.8497
C16—C17	1.378 (4)	O2'—H2'B	0.8501
C16—H16A	0.9300	O1'—H1'A	0.8498
C17—C18	1.356 (5)	O1'—H1'B	0.8500
C17—H17A	0.9300		
			
O3—P1—C15	110.21 (10)	C18—C17—C16	120.6 (3)
O3—P1—C21	112.49 (10)	C18—C17—H17A	119.7
C15—P1—C21	106.11 (11)	C16—C17—H17A	119.7
O3—P1—C2	115.74 (10)	C19—C18—C17	119.0 (3)
C15—P1—C2	104.62 (11)	C19—C18—H18A	120.5
C21—P1—C2	106.96 (10)	C17—C18—H18A	120.5
O4—P2—C33	111.83 (12)	C18—C19—C20	121.3 (3)
O4—P2—C27	110.74 (10)	C18—C19—H19A	119.3
C33—P2—C27	106.64 (11)	C20—C19—H19A	119.3
O4—P2—C8	115.67 (10)	C15—C20—C19	120.6 (3)
C33—P2—C8	107.27 (11)	C15—C20—H20A	119.7
C27—P2—C8	104.04 (10)	C19—C20—H20A	119.7
C6—O1—C14	118.5 (2)	C26—C21—C22	117.8 (2)
C12—O2—C13	119.0 (2)	C26—C21—P1	124.26 (18)
C2—C1—C6	118.86 (19)	C22—C21—P1	117.94 (18)
C2—C1—C7	122.79 (18)	C23—C22—C21	120.9 (2)
C6—C1—C7	118.35 (19)	C23—C22—H22A	119.5
C1—C2—C3	119.3 (2)	C21—C22—H22A	119.5
C1—C2—P1	122.21 (15)	C24—C23—C22	119.9 (3)
C3—C2—P1	118.48 (18)	C24—C23—H23A	120.1
C4—C3—C2	120.6 (2)	C22—C23—H23A	120.1
C4—C3—H3A	119.7	C25—C24—C23	120.2 (3)
C2—C3—H3A	119.7	C25—C24—H24A	119.9
C3—C4—C5	120.8 (2)	C23—C24—H24A	119.9
C3—C4—H4A	119.6	C24—C25—C26	120.1 (2)
C5—C4—H4A	119.6	C24—C25—H25A	119.9
C6—C5—C4	119.3 (2)	C26—C25—H25A	119.9
C6—C5—H5A	120.4	C21—C26—C25	121.0 (2)
C4—C5—H5A	120.4	C21—C26—H26A	119.5
O1—C6—C5	123.9 (2)	C25—C26—H26A	119.5
O1—C6—C1	114.99 (19)	C28—C27—C32	119.0 (2)
C5—C6—C1	121.1 (2)	C28—C27—P2	116.79 (17)
C8—C7—C12	119.0 (2)	C32—C27—P2	124.16 (19)
C8—C7—C1	122.63 (18)	C29—C28—C27	120.4 (2)
C12—C7—C1	118.33 (19)	C29—C28—H28A	119.8
C7—C8—C9	119.4 (2)	C27—C28—H28A	119.8
C7—C8—P2	121.80 (15)	C30—C29—C28	120.1 (2)
C9—C8—P2	118.85 (18)	C30—C29—H29A	119.9
C10—C9—C8	120.2 (2)	C28—C29—H29A	119.9
C10—C9—H9A	119.9	C31—C30—C29	120.2 (2)
C8—C9—H9A	119.9	C31—C30—H30A	119.9
C9—C10—C11	120.9 (2)	C29—C30—H30A	119.9
C9—C10—H10A	119.6	C30—C31—C32	120.6 (2)
C11—C10—H10A	119.6	C30—C31—H31A	119.7
C10—C11—C12	119.8 (2)	C32—C31—H31A	119.7
C10—C11—H11A	120.1	C31—C32—C27	119.7 (2)
C12—C11—H11A	120.1	C31—C32—H32A	120.1
O2—C12—C11	124.0 (2)	C27—C32—H32A	120.1
O2—C12—C7	115.2 (2)	C34—C33—C38	118.1 (3)
C11—C12—C7	120.8 (2)	C34—C33—P2	117.6 (3)
O2—C13—H13A	109.5	C38—C33—P2	124.3 (2)
O2—C13—H13B	109.5	C33—C34—C35	120.1 (4)
H13A—C13—H13B	109.5	C33—C34—H34A	120.0
O2—C13—H13C	109.5	C35—C34—H34A	120.0
H13A—C13—H13C	109.5	C36—C35—C34	121.8 (4)
H13B—C13—H13C	109.5	C36—C35—H35A	119.1
O1—C14—H14A	109.5	C34—C35—H35A	119.1
O1—C14—H14B	109.5	C35—C36—C37	119.0 (4)
H14A—C14—H14B	109.5	C35—C36—H36A	120.5
O1—C14—H14C	109.5	C37—C36—H36A	120.5
H14A—C14—H14C	109.5	C36—C37—C38	120.1 (4)
H14B—C14—H14C	109.5	C36—C37—H37A	120.0
C16—C15—C20	117.4 (2)	C38—C37—H37A	120.0
C16—C15—P1	124.7 (2)	C33—C38—C37	121.0 (3)
C20—C15—P1	117.90 (19)	C33—C38—H38A	119.5
C15—C16—C17	121.1 (3)	C37—C38—H38A	119.5
C15—C16—H16A	119.5	H2'A—O2'—H2'B	104.8
C17—C16—H16A	119.5	H1'A—O1'—H1'B	103.5
			
C6—C1—C2—C3	0.0 (3)	C21—P1—C15—C20	−170.98 (19)
C7—C1—C2—C3	179.25 (19)	C2—P1—C15—C20	76.1 (2)
C6—C1—C2—P1	178.48 (16)	C20—C15—C16—C17	−1.6 (6)
C7—C1—C2—P1	−2.3 (3)	P1—C15—C16—C17	−178.9 (4)
O3—P1—C2—C1	−24.2 (2)	C15—C16—C17—C18	1.8 (8)
C15—P1—C2—C1	−145.72 (17)	C16—C17—C18—C19	−0.6 (7)
C21—P1—C2—C1	101.98 (18)	C17—C18—C19—C20	−0.6 (6)
O3—P1—C2—C3	154.22 (17)	C16—C15—C20—C19	0.4 (4)
C15—P1—C2—C3	32.7 (2)	P1—C15—C20—C19	177.9 (2)
C21—P1—C2—C3	−79.55 (19)	C18—C19—C20—C15	0.7 (5)
C1—C2—C3—C4	−0.5 (3)	O3—P1—C21—C26	133.4 (2)
P1—C2—C3—C4	−178.99 (19)	C15—P1—C21—C26	−106.0 (2)
C2—C3—C4—C5	0.9 (4)	C2—P1—C21—C26	5.3 (2)
C3—C4—C5—C6	−0.9 (4)	O3—P1—C21—C22	−46.7 (2)
C14—O1—C6—C5	−10.7 (4)	C15—P1—C21—C22	73.9 (2)
C14—O1—C6—C1	169.9 (2)	C2—P1—C21—C22	−174.8 (2)
C4—C5—C6—O1	−178.9 (2)	C26—C21—C22—C23	1.2 (4)
C4—C5—C6—C1	0.4 (4)	P1—C21—C22—C23	−178.7 (3)
C2—C1—C6—O1	179.40 (19)	C21—C22—C23—C24	−0.7 (5)
C7—C1—C6—O1	0.1 (3)	C22—C23—C24—C25	0.0 (5)
C2—C1—C6—C5	0.0 (3)	C23—C24—C25—C26	0.2 (4)
C7—C1—C6—C5	−179.3 (2)	C22—C21—C26—C25	−1.1 (4)
C2—C1—C7—C8	96.8 (3)	P1—C21—C26—C25	178.8 (2)
C6—C1—C7—C8	−84.0 (3)	C24—C25—C26—C21	0.4 (4)
C2—C1—C7—C12	−84.4 (3)	O4—P2—C27—C28	−53.6 (2)
C6—C1—C7—C12	94.8 (2)	C33—P2—C27—C28	−175.45 (19)
C12—C7—C8—C9	0.9 (3)	C8—P2—C27—C28	71.3 (2)
C1—C7—C8—C9	179.64 (19)	O4—P2—C27—C32	123.6 (2)
C12—C7—C8—P2	−179.68 (16)	C33—P2—C27—C32	1.7 (2)
C1—C7—C8—P2	−0.9 (3)	C8—P2—C27—C32	−111.5 (2)
O4—P2—C8—C7	−27.9 (2)	C32—C27—C28—C29	−0.8 (4)
C33—P2—C8—C7	97.62 (19)	P2—C27—C28—C29	176.4 (2)
C27—P2—C8—C7	−149.62 (17)	C27—C28—C29—C30	0.3 (4)
O4—P2—C8—C9	151.54 (17)	C28—C29—C30—C31	0.6 (4)
C33—P2—C8—C9	−82.90 (19)	C29—C30—C31—C32	−0.9 (4)
C27—P2—C8—C9	29.9 (2)	C30—C31—C32—C27	0.2 (4)
C7—C8—C9—C10	0.1 (3)	C28—C27—C32—C31	0.6 (4)
P2—C8—C9—C10	−179.40 (18)	P2—C27—C32—C31	−176.5 (2)
C8—C9—C10—C11	−0.3 (4)	O4—P2—C33—C34	−25.2 (3)
C9—C10—C11—C12	−0.5 (4)	C27—P2—C33—C34	96.0 (3)
C13—O2—C12—C11	4.0 (4)	C8—P2—C33—C34	−153.0 (3)
C13—O2—C12—C7	−175.4 (3)	O4—P2—C33—C38	154.3 (2)
C10—C11—C12—O2	−177.9 (2)	C27—P2—C33—C38	−84.5 (3)
C10—C11—C12—C7	1.4 (4)	C8—P2—C33—C38	26.4 (3)
C8—C7—C12—O2	177.8 (2)	C38—C33—C34—C35	2.1 (6)
C1—C7—C12—O2	−1.1 (3)	P2—C33—C34—C35	−178.4 (4)
C8—C7—C12—C11	−1.6 (3)	C33—C34—C35—C36	−1.9 (8)
C1—C7—C12—C11	179.5 (2)	C34—C35—C36—C37	1.1 (8)
O3—P1—C15—C16	128.4 (3)	C35—C36—C37—C38	−0.6 (7)
C21—P1—C15—C16	6.4 (3)	C34—C33—C38—C37	−1.6 (5)
C2—P1—C15—C16	−106.5 (3)	P2—C33—C38—C37	178.9 (3)
O3—P1—C15—C20	−48.9 (2)	C36—C37—C38—C33	0.9 (6)

### Hydrogen-bond geometry (Å, º)

**Table:**

*D*—H···*A*	*D*—H	H···*A*	*D*···*A*	*D*—H···*A*
O1′—H1′*A*···O4	0.85	2.31	2.854 (4)	123
O2′—H2′*A*···O3	0.85	1.96	2.799 (3)	169
